# Diabetes Insipidus Complicating Diabetes Mellitus Type 1: A Pituitary Abscess Diagnosis

**DOI:** 10.1210/jcemcr/luae057

**Published:** 2024-06-03

**Authors:** Barbara Lionetti, Nicola Minuto, Marta Bassi, Flavia Napoli

**Affiliations:** Department of Neurosciences, Rehabilitation, Ophthalmology, Genetics, Maternal and Child Health, University of Genoa, 16132, Genoa, Italy; Pediatric Clinic and Endocrinology Unit, IRCCS Istituto Giannina Gaslini, 16147, Genoa, Italy; Pediatric Clinic and Endocrinology Unit, Regional Center for Pediatric Diabetes, IRCCS Istituto Giannina Gaslini, 16147, Genoa, Italy; Department of Neurosciences, Rehabilitation, Ophthalmology, Genetics, Maternal and Child Health, University of Genoa, 16132, Genoa, Italy; Pediatric Clinic and Endocrinology Unit, IRCCS Istituto Giannina Gaslini, 16147, Genoa, Italy; Pediatric Clinic and Endocrinology Unit, Regional Center for Pediatric Diabetes, IRCCS Istituto Giannina Gaslini, 16147, Genoa, Italy; Pediatric Clinic and Endocrinology Unit, IRCCS Istituto Giannina Gaslini, 16147, Genoa, Italy; Pediatric Clinic and Endocrinology Unit, Regional Center for Pediatric Diabetes, IRCCS Istituto Giannina Gaslini, 16147, Genoa, Italy

**Keywords:** type 1 diabetes mellitus, diabetes insipidus, hypopituitarism, infections, pituitary abscess

## Abstract

In this report we present a case of a 14-year-old girl with type 1 diabetes mellitus (T1DM) who experienced glycemic instability and multiple hormonal deficits, including diabetes insipidus, central hypothyroidism, and central adrenal insufficiency. Brain and sellar magnetic resonance imaging revealed a mass in the suprasellar region, which was confirmed to be a pituitary abscess through transsphenoidal biopsy.

T1DM is a chronic systemic disease that can lead to suboptimal glycemic control and increased susceptibility to infections. Pituitary abscess is a rare and serious infection that can manifest with nonspecific signs and symptoms, as well as pituitary hormonal deficiencies.

Currently, after a 6-year follow-up the pituitary hormone deficiencies have resolved apart from persistent partial diabetes insipidus. Through a review of the current literature, we discuss the clinical characteristics of pituitary abscess, the challenges in diagnosing it, and speculate on the potential clinical and pathophysiological relationship between this uncommon infection and T1DM in our patient.

## Introduction

We present a case of a patient with type 1 diabetes mellitus (T1DM) who developed polyuria and polydipsia, along with other nonspecific symptoms. These symptoms occurred 7 years after the onset of T1DM. Further evaluation demonstrated multiple pituitary hormonal deficits, including diabetes insipidus, hypogonadism, hypothyroidism, and central adrenal insufficiency. Brain imaging revealed a sellar-suprasellar mass, which was found to be a pituitary abscess. In this report, we have explored the potential clinical and pathophysiological relationship between this rare infection and T1DM, as well as the potential challenge of identifying diabetes insipidus and adrenal insufficiency in a patient with T1DM.

## Case Presentation

In 2017, a 14-year-old girl with T1DM since age 7 years was admitted to the Pediatric Endocrinology and Diabetology department in Genoa, Italy, because of a 2-month history of progressive asthenia, weight loss, headaches, and glycemic instability with multiple hypoglycemic episodes. She also reported increased urine output and polydipsia during the previous 2 weeks, without significant correlation to hyperglycemia. At the time of admission, the patient's daily water input was 5 to 6 L of water and there was a 2-month history of secondary amenorrhea. The past medical history of the patient included appendectomy at age 8 for acute perforated appendicitis and chronic autoimmune thyroiditis with normal thyroid function. She was undergoing multiple daily injection basal-bolus insulin therapy, and her daily insulin requirement was 0.8 U/kg/day. Physical examination displayed a Tanner stage 5 both for pubic hair and breast development, height was 159.8 cm (48th percentile of the World Health Organization growth charts), and weight was 51.7 kg (55th percentile of the World Health Organization growth charts). Vital signs and medical evaluation, as well as neurological examination and examination of the fundus oculi, were normal.

## Diagnostic Assessment

The initial workup included a chest x-ray, an abdominal ultrasound, and an electrocardiogram, all of which showed no pathological findings. Laboratory evaluation revealed satisfactory glucometabolic control (glycated hemoglobin A_1c_, 7.18%, 54 mmol/mol normal range (n.r.): 20-42 mmol/mol), a modest reduction in hemoglobin (11.6 g/dL, 116 g/L, n.r.: 12-16.5 g/dL, 120-155 g/dL), normal levels of serum electrolytes, liver function, and lipid profile. Inflammatory markers were negative.

A hormonal laboratory workup showed multiple pituitary hormonal deficits. Baseline evaluation of the adrenal axis at 8 Am showed adrenocorticotropin (ACTH) and cortisol levels at the lower limit of the normal range (Table 1). A low-dose ACTH stimulation test confirmed the diagnosis of central adrenal insufficiency, with a peak cortisol value of 338.37 nmol/L (12.26 μg/dL) [1]. Central hypothyroidism was detected through a modest decrease of free thyroxine and a decrease of thyrotropin (see Table 1). The demonstration of polyuria and polydipsia, unrelated to capillary blood glucose levels, through water input and output monitoring, was used together with urinary osmolarity of 59 mOsm/kg (59 mmol/kg) as diagnostic criteria to diagnose diabetes insipidus [2]. A water deprivation test was not performed due to the concomitance of other pituitary hormonal deficits. Hypogonadism was confirmed by undetectable 17-β-estradiol levels. Basal prolactin was elevated: 89.6 ng/mL (89.6 µg/L [n.r. 4.8-23.3 ng/mL]). Measurements were similar 30 and 60 minutes after positioning a peripheral venous catheter to remove the effect of emotional stress related to pain and venipuncture: basal 89.6 ng/mL (89.6 µg/L); 30 minutes: 91 ng/mL (91 µg/L); 60 minutes: 93.9 ng/mL (93.9 µg/L).

**Table 1.** Blood laboratory tests on admission and at last follow-up

	On admission	Follow-up at 5 years	Normal values
Cortisol	3.81 μg/dL (104.97 nmol/L)	14.00 μg/dL (386.26 nmol/L)	2.47-19.50 μg/dL (68.10-538.0 nmol/L)
ACTH	13.94 pg/mL (3.07 pmol/L)	5.77 pg/mL (1.27 pmol/L)	7.20-63.3 pg/mL (1.58-13.92 pmol/L)
TSH	0.03 μUI/mL (0.09 mU/L)	2.21 μUI/mL (5.52 mU/L)	0.20-4.20 μUI/mL (0.50-10.5 mU/L)
Free T3	4.30 pg/mL (n.v. 2.0-4.40 pg/mL) 68.60 pmol/L (n.v. 32.0-70.50 pmol/L)	2.60 pg/mL (n.v. 3.10-4.80 pg/mL) 40.64 pmol/L (n.v. 47.70-73.90 pmol/L)	
Free T4	0.91 ng/dL (n.v. 0.93-1.70 ng/dL), 117.05 pmol/L (n.v. 119.69-218.79 pmol/L)	1.10 ng/dL (n.v. 0.9-1.53 ng/dL), 141.57 pmol/L (n.v. 115.80-196.90 pmol/L)	
LH	0.40 mIU/mL (0.40 UI/L)	8.80 mIU/mL (8.80 UI/L)	Premenopausal women: 5-25 mIU/mL(5-25 IU/L)
FSH	3.30 mIU/mL (3.30 UI/L)	7.10 mIU/mL (7.10 UI/L)	Premenopausal women: 4.7-21.5 mIU/mL (4.5-21.5 IU/L)
17-β-estradiol	<5 pg/mL (<18.35 pmol/L)	77 pg/mL (282.59 pmol/L)	Premenopausal women: 30-400 pg/mL (110-1468.40 pmol/L)
Na^+^	141 mEq/L (141 mmol/L)	136 mEq/L (136 mmol/L)	135-145 mEq/L (135-145 mmol/L)
Urine osmolality	59 mOsm/kg H_2_O 59 mmol/kg	637 mOsm/kg H_2_O 637 mmol/kg	300-900 mOsm/kg H_2_O 300-900 mmol/kg
Serum osmolality	286 mOsm/kg H_2_O 286 mmol/kg	285 mOsm/kg H_2_O 285 mmol/kg	275-295 mOsm/kg H_2_O 275-294 mmol/kg

Abbreviations: ACTH, adrenocorticotropin; CRP, C-reactive protein; ERS, erythrocyte sedimentation rate; FSH, follicular-stimulating hormone; GH, growth hormone, IGF-1 insulin-like growth factor-1; LH, luteinizing hormone; n.v., normal values; T3, 3,5,3′-triiodothyronine; T4, thyroxine; TSH, thyrotropin.

**Figure 1.**
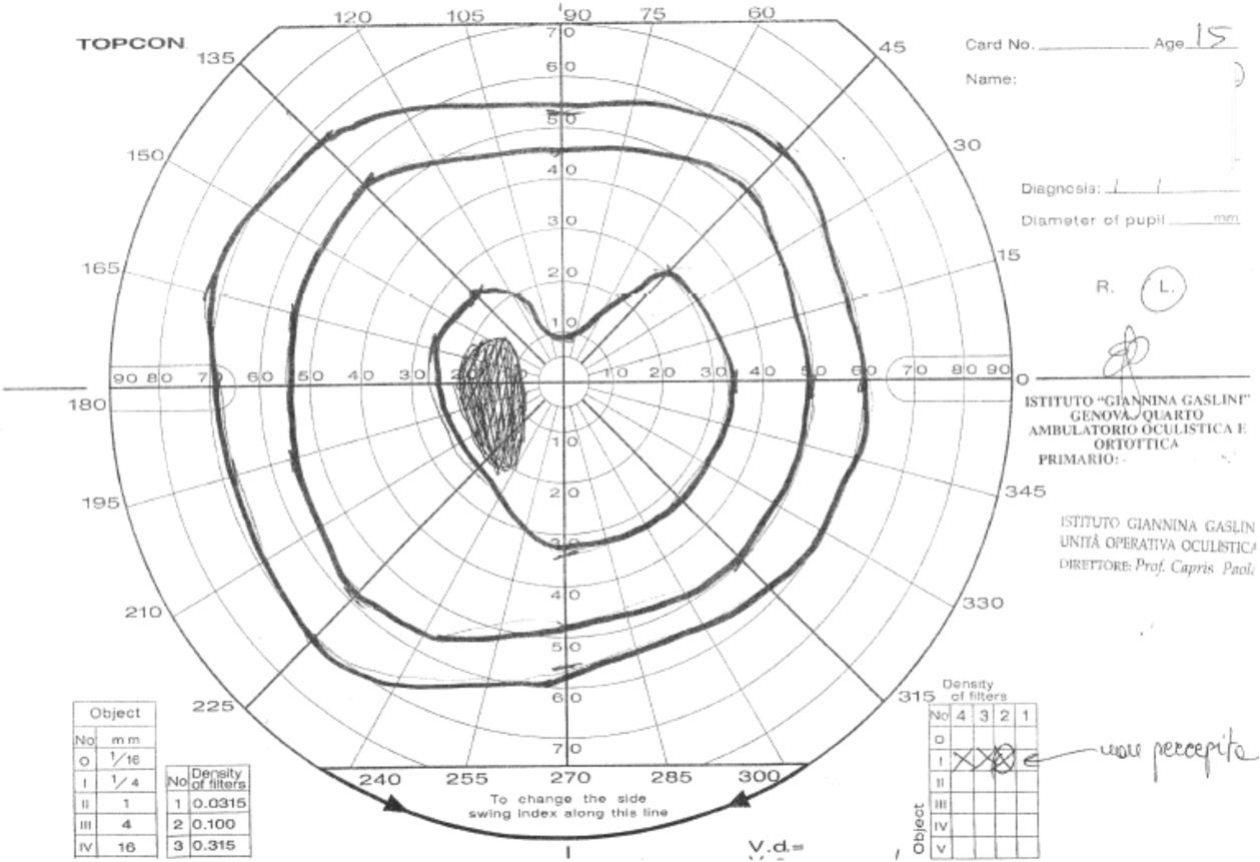
The first magnetic resonance imaging examination confirmed a solid sellar-suprasellar expansive process (A, 3D T2-DRIVE sagittal sequence; B, T1-weighted sequence) with C and D, postcontrast enhancement; central necrotic area without intralesional bleeding; additionally, a thickening of the pituitary stalk and the absence of the normal posterior pituitary bright spot were observed. The computed tomography scan confirmed a widened appearance of the sella turcica due to the sellar-suprasellar lesion, which presented inhomogeneous intensity due to hypodense central necrosis.

**Figure 2.**
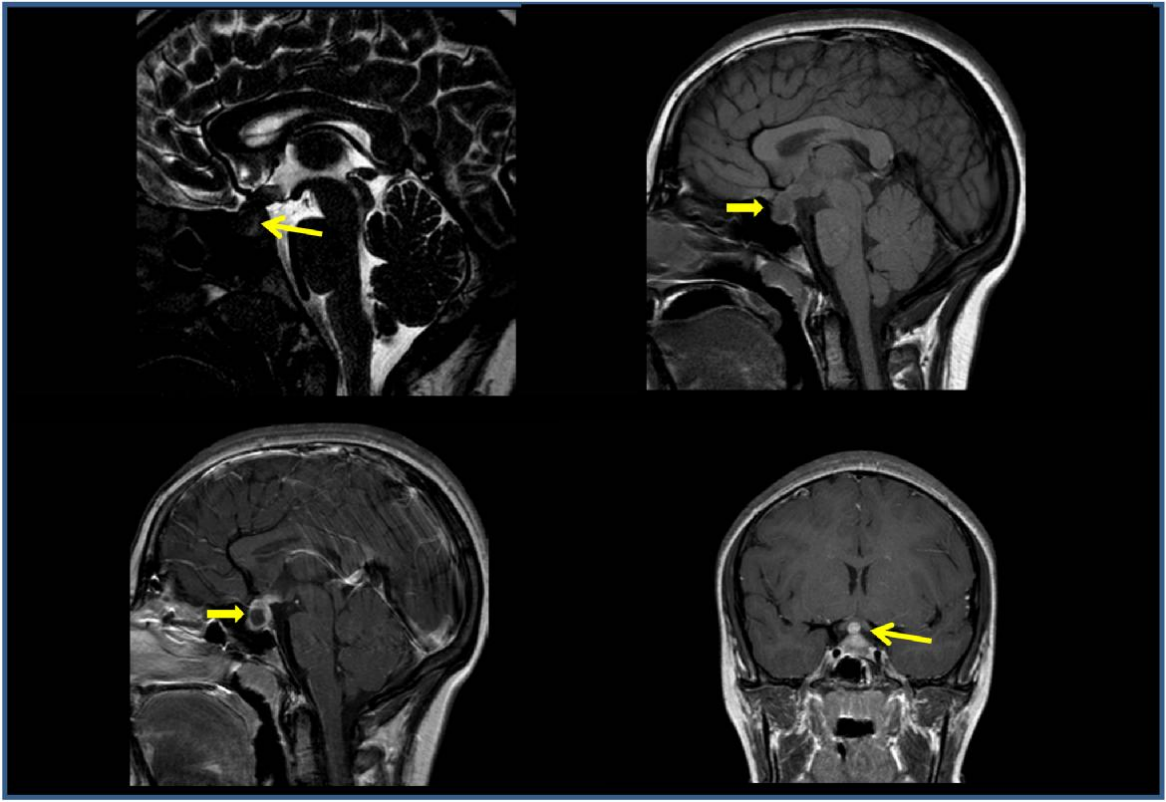
The visual field examination revealed an incision in the upper sector of the central isopter and an enlargement of the blind spot.

Infectious disease screening including toxoplasma, rubeola, cytomegalovirus, Epstein Barr virus, herpes simplex, and parvovirus serology, multiplex polymerase chain reaction for HHV6 on blood and respiratory bacteria and viruses on nasopharyngeal swabs were negative. Cerebrospinal fluid chemical and cytological examinations from lumbar puncture were normal. The visual field examination revealed an incision of the central isopter in the upper sector and enlargement of the blind spot (Fig. 1). Magnetic resonance imaging (MRI) (Fig. 2) showed a solid sellar-suprasellar expansive process with postcontrast enhancement, central necrotic area without intralesional bleeding, thickening of the pituitary stalk, and the absence of the normal posterior pituitary bright spot.

Differential diagnoses included Langerhans cell histiocytosis, hypophyseal adenoma, pituitary inflammation, germinoma, although the central necrotic component is not common in this condition, and pituitary apoplexy, although the lack of the posterior pituitary bright spot and diabetes insipidus are not typically associated with this condition. To rule out neoplastic involvement serum levels of α-fetoprotein, β-human chorionic gonadotropin, and neuron-specific enolase were carried out with negative results. To investigate possible multiple localizations of Langerhans cells histiocytosis or other metastatic disease, a whole-body short tau inversion recovery (STIR) MRI was performed, and it yielded negative results.

Finally, a transsphenoidal biopsy of the lesion was performed. During the procedure, draining of purulent material was observed. The bacterial culture test of the sample was positive for *Staphylococcus aureus* and *Streptococcus salivaris*, leading to a diagnosis of pituitary abscess.

## Treatment

Oral hydrocortisone replacement therapy was initiated (10 mg/m^2^ in 3 daily doses), as well as desmopressin sublingual tablets with a dose of 15 mcg every 8 hours tapered according to serum sodium levels. Oral L-thyroxine was initiated at a dose of 25 mcg every 24 hours. Subcutaneous insulin continued to be administered with multiple daily injections. After the biopsy, intravenous ceftriaxone, metronidazole, and trimethoprim/sulfamethoxazole were initiated. After 8 days from the start of antibiotics, the patient developed a macular erythematous rash unresponsive to antihistamine therapy, hyperpyrexia, and an increase in inflammatory markers. Ceftriaxone was replaced with teicoplanin, and the dose of hydrocortisone was doubled to the stress dose of 20 mg/m^2^/day orally.

During the following days, the patient developed an allergic reaction with severe hypotension with bradycardia, generalized urticaria, further increase in inflammatory markers, and leukopenia. Teicoplanin was therefore switched to clindamycin, and high-dose intravenous steroids were started, with good clinical response.

## Outcome and Follow-up

The patient was dismissed after 1 month in good clinical condition. After 6 years of follow-up, at the time of this writing, she remains clinically well with good glycemic control. Most of the hormonal pituitary deficits have resolved, but she continues to demonstrate partial diabetes insipidus treated with sublingual desmopressin (15 mcg every 24 hours). Follow-up MRI was performed in 2018 and 2019, showing a complete resolution of the structural pituitary abnormalities.

## Discussion

Pituitary abscess is a rare condition, accounting for 0.2% to 1% of pituitary lesions. It can be either primary or secondary arising from within a preexisting pituitary lesion such as craniopharyngioma, adenoma, or Rathke cleft cyst. Spreading can occur by contiguity in the presence of meningitis or sinusitis, or by a hematogenous route. Causative agents responsible can be both bacteria and fungi, although most of the cultures of material drained from pituitary abscesses have been reported to be sterile [3]. In the case reported, our patient exhibited no apparent structural risk factors and the MRI showed no signs of sinusitis or preexisting pituitary lesions.

Zhang et al [4] reviewed 29 cases of pituitary abscess whose most common complaints were headache (21 cases, 72.4%), with no peculiar pattern, hypodynamia, somnolence (15 cases, 51.7%), and visual disturbances (10 cases, 34.5%). Nineteen patients presented with anterior pituitary hypofunction (65.5%), and 12 patients (41.4%) had diabetes insipidus. Other symptoms included mild or moderate fever, dizziness, or tinnitus. A history of paranasal sinusitis was found in 6 cases out of 29.

Pituitary abscess diagnosis remains challenging, not only due to the lack of specific symptoms but also because of its radiological features, which are often inconclusive. Typically, an etiological diagnosis is not achieved even after clinical, biochemical, and radiological evaluation, but it is attained only through histology. In a systematic review by Shkarubo et al [5] of 41 cases of abscesses in this region, a primary abscess was suspected in only 54.8% of patients before surgery. Differential diagnoses include pituitary adenomas and craniopharyngiomas or other pituitary tumors.

Broad-spectrum antibiotics and surgical drainage, through a transsphenoidal approach, are the first line of treatment, though surgery itself may perpetuate or cause anterior pituitary insufficiency and diabetes insipidus if the gland is damaged. It is indeed common to observe residual hormonal deficits (Liu et al [6] reported that 22 out of 30 patients continued to require hormone replacement therapy).

Review of the available literature reveals that pituitary abscess in T1DM has been reported in very few cases, and the risk of occurrence in this population seems anecdotal [7]. Despite the absence of a clear etiological link in the reported case and the available literature, T1DM remains the only risk factor known to us for the development of this rare infectious condition present in the patient at the time of diagnosis. Although it is commonly believed that people with T1DM are more susceptible to infectious diseases, few studies report on infection outcomes among this population. Those available describe an increased susceptibility and mortality from infectious diseases in comparison to the general population. One of the largest studies to date on infections in T1DM used the Australian diabetes register and showed elevated mortality from septicemia and osteomyelitis among individuals with T1DM [8]. DM is frequently associated with an impaired immune response against infectious agents due to microangiopathy and direct impairment of the function of immune system cells, putting affected patients at risk for more severe infectious disease courses, especially those with poorer glycemic control or carrying comorbidities [9].

We believe our case to be instructional not only because it led to the rare diagnosis of pituitary abscess, but also because of the coexistence of T1DM and multiple pituitary hormone deficiencies whose clinical features may overlap. To demonstrate the etiology of polyuria and polydipsia, close glucose monitoring and water input and output monitoring had to be compared. Moreover, hypoglycemia caused by low cortisol levels could have been disguised as iatrogenic hypoglycemia, which is frequent in patients with T1DM. Although the patient did not use continuous glucose monitoring (CGM) at the time of disease presentation, it may have helped to accelerate the diagnosis: In 2021 a case reported the diagnosis of adrenal insufficiency suggested by glycemic instabilities observed through CGM [10].

In conclusion, pituitary abscesses are severe infectious diseases, often without substantial history or symptoms. The clinical course of pituitary abscess can be complicated, and hormonal deficits can perpetuate after treatment of the underlying disease, therefore multidisciplinary management and careful follow-up are needed. We cannot irrefutably correlate T1DM with the development of a pituitary abscess in our patient, although DM appears to increase susceptibility to infectious diseases and was the only known risk factor present in our case.

## Learning Points

- Pituitary abscess is a rare condition, and diagnosis remains challenging due to a lack of specific symptoms and radiological features: Abscesses, adenomas, and other neoplastic entities may overlap.
- The presence of multiple hormonal deficits consistent with a secondary etiology suggests that diagnostic imaging of the pituitary gland should be undertaken.
- Glycemic instability in people with T1DM is common. Although usually related to insulin therapy, it can be the first sign of ongoing infection, systemic inflammation, and in rarer instances, pituitary hormonal defects.
- A history of polyuria and polydipsia caused by diabetes insipidus can be difficult to recognize in patients with T1DM.

## Contributors

All authors made individual contributions to authorship. F.N. and N.M. were involved in the diagnosis and management of this patient and manuscript submission. M.B. and B.L. were responsible for the drafting of this case report. All authors reviewed and approved the final draft.

## Data Availability

Original data generated and analyzed during this study are included in this published article.

## Abbreviations

ACTH: adrenocorticotropin
CGM: continuous glucose monitoring
MRI: magnetic resonance imaging
T1DM: type 1 diabetes mellitus
TSH: thyrotropin
